# Wild *Myrtus communis* L. Fruit By-Product as a Promising Source of a New Natural Food Colourant: Optimization of the Extraction Process and Chemical Characterization

**DOI:** 10.3390/foods14030520

**Published:** 2025-02-06

**Authors:** Erika N. Vega, Lorena González-Zamorano, Elena Cebadera, Lillian Barros, Tayse F. F. da Silveira, Guillermo Vidal-Diez de Ulzurrun, Javier Tardío, Almudena Lázaro, Montaña Cámara, Virginia Fernández-Ruíz, Patricia Morales

**Affiliations:** 1 Departamento Nutrición y Ciencia de los Alimentos, Facultad de Farmacia, Universidad Complutense de Madrid, Plaza Ramón y Cajal, s/n, 28040 Madrid, Spain; erinino@ucm.es (E.N.V.); lorego06@ucm.es (L.G.-Z.); ecebadera@farm.ucm.es (E.C.); mcamara@ucm.es (M.C.); vfernand@farm.ucm.es (V.F.-R.); 2 Centro de Investigação de Montanha (CIMO), LA SusTEC Instituto Politécnico de Bragança, Campus de Santa Apolónia, 5300-253 Bragança, Portugal; lillian@ipb.pt (L.B.); tayse.silveira@ipb.pt (T.F.F.d.S.); 3 Institute of Molecular Biology, Academia Sinica, Nangang, 128 Academia Road, Section 2, Taipei 115, Taiwan; gvidal@gate.sinica.edu.tw; 4 Instituto Madrileño de Investigación y Desarrollo Rural, Agrario y Alimentario (IMIDRA), Finca “El Encín”, Apdo. 127, 28800 Alcalá de Henares, Spain; javier.tardio@madrid.org (J.T.); almudena.lazaro@madrid.org (A.L.)

**Keywords:** natural colourant, anthocyanins, bioactive compounds, Mirtle, extraction optimization

## Abstract

*Myrtus communis* L., as a wild underutilized fruit, was analyzed for its physicochemical properties and bioactive composition, revealing a high anthocyanin content principally concentrated in the peel. Therefore, the anthocyanin extraction conditions through ultrasound-assisted extraction from *Myrtus communis* L. fruit peels (MCP), considered a by-product, were optimized using response surface methodology (RSM), evaluating four independent extraction variables with total anthocyanin content as the response criterion. As a result, optimal extraction conditions were determined to be 20 min, pH 6, 500 W, and 19.68 g/L, yielding a total anthocyanin content of 47.51 mg cya-3-glu/g. In addition, the optimized colourant extract presented a higher content of bioactive compounds compared to the fruit itself, with 1.4 times higher polyphenols and 1.8 times higher total anthocyanin content, with malvidin-3-*O*-glucoside as the predominant anthocyanin, evidencing the effectiveness of the proposed extraction process. In conclusion, applying the optimal extraction conditions for MPC enables the production of an extract with remarkable anthocyanin content and other phenolic compounds, making it an excellent candidate as a natural food colourant.

## 1. Introduction

Colourants are additives widely used by the food industry, applied to enhance, improve or restore the colour lost during the production process. They have become one of the principal additives since colour is a decisive parameter in the acceptance or rejection of a food product; colour can generate an idea of its taste, freshness, or composition [1,2,3]. Colourants can be classified as artificial, generated synthetically, and natural, obtained from vegetables, animals, or minerals; the former being the most used by the food industry due to their high stability, strong colour, and low prices. However, in recent years, different research has evidenced the possible link between artificial colourants, especially the azo ones, with urticaria, allergies, and, in the worst cases, attention deficit and hyperactivity disorder (ADHD), evidenced mostly in kids [4,5,6,7].

In addition to the evidence of side effects due to the consumption of artificial colourants, awareness among human beings regarding the importance and influence that food has on their well-being has been increasing. This led to the demand of healthier food products, made without additives, or at least with natural ones that do not generate side effects [8].

As a result of the above, research on natural colourants has become an important field, covering the search for new sources or matrices for natural colourant obtention or extraction, their characterization, the optimization of their extraction from each matrix, to the improvement of their stability through different methods as encapsulation, co-pigmentation among others. The principal problem with natural colourants is that their stability can be affected by different factor such as pH, light, and the presence of oxygen, among others [2,9]. The principal natural colourants are based in anthocyanins, carotenoids, betalains, or chlorophylls, the former being the most abundant and consequently the most studied, with more than 700 different anthocyanins reported to date [10,11,12]. However, natural colourants based on anthocyanins exhibit high instability to factors such as temperature and light, resulting in their degradation and loss of colouration. In addition, their colour is largely dependent on the pH of the medium, with most colourants based on anthocyanins producing shades of red. These limitations drive the ongoing search for new sources of anthocyanins that offer improved stability and a broader range of colours [10,13].

Myrtle (*Myrtus communis* L.) is an evergreen shrub from the Myrtaceae family, native to Mediterranean countries, typically growing 1 to 5 m tall. This shrub features abundant branches with green lanceolate leaves, white flowers, and blueish-black berry fruits, up to 1 cm, with whitish pulp and light brown seeds. In Spain and other Mediterranean countries, *M. communis* has been used traditionally as a spice, in the production of alcoholic beverages and for medicinal purposes, such as antidiarrheic, anti-inflammatory, and for the treatment of skin allergies and respiratory infections [14,15]. This species has been widely studied, principally regarding its essential oil composition due to its importance in the perfume industry, where myrtenyl acetate, limonene + 1,8-cineole, α-pinene, and linalool have been reported as the principal essential oils found in the fruit [14,16]. Additionally, *M. communis* fruits present a rich composition, containing representative amounts of dietary fibre (17.4 g/100 g), proteins (1.66–4.17 g/100 g), and minerals like iron (1.60–2.56 mg/100 g) and potassium (478–549.9 mg/100 g) [15,17], as well as different bioactive compounds, with total phenolic content ranging between 88.47 and 48.96 mg/100 g and anthocyanins ranging from 4.07 to 242 mg/100 g [18]. Due to its rich composition, different biological activities have been linked to *M. communis* fruits, such as anti-inflammatory, antiseptic, antimicrobial, and hypoglycaemic [19,20].

The growing demand for natural colourants has led to the development of different extraction methodologies such as maceration [21], ultrasound-assisted extraction (UAE) [22,23], microwave-assisted extraction (MAE) [24], and supercritical fluid extraction (SFE) [25], among others. Among these, UAE has emerged as a standout technique due to its consistently excellent results. For example, UAE has demonstrated superior performance in extracting anthocyanins from grape pomace when compared to maceration [26], possibly because maceration often requires high temperatures that can degrade anthocyanins. Similarly, UAE has outperformed MAE in extracting anthocyanins from common beans (*Phaseolus vulgaris* L.) yielding higher concentrations [27].

UAE has stood out for its work mechanism based on cavitation, where the ultrasound waves allow the liberation of the colourant molecules by generating the rupture of the tissue. This approach allows superior anthocyanin extraction within significantly shorter times compared to other methods, while also reducing the use of solvents and aligning with the principles of green chemistry [28,29]. While some studies have examined extraction methods for *M. communis* (e.g., MAE) to better characterize its bioactive compounds and essential oils, no optimization has been performed to develop a natural food colourant candidate [30,31,32].

In response to the problems linked to the use of artificial colourants and the rising demand for natural food colourants and healthier food products, this study aims to determine the optimal extraction conditions for anthocyanins from *M. communis* using response surface methodology (RSM) to generate a candidate of novel food natural colourant, while also characterizing the fruit and optimized extract in terms of physicochemical properties and bioactive compounds. Both the extraction conditions and the chemical composition are protected by a Spanish patent application that has been published under number ES2990137A1, filed at the Spanish Patent Office.

## 2. Materials and Methods

### 2.1. Samples

Mature fruits of *M. communis* L. were harvested in Spain at two different locations during 2021 and 2022 (Table 1), following the harvesting permission for 2021 Ref. PN-NC_032021 and Ref. ABSCH-IRCC-ES-257749-1, and for 2022 Ref. PN-NC_022022 and Ref. ABSCH-IRCC-ES-262067-1 issued by the Spain Ministry of Agriculture, Fishier and Food (MAPA). In a representative amount of the fruits, the physicochemical (moisture, pH and titratable acidity) characterization was carried out. The peel, treated as a by-product, was separated from the rest of the fruit, frozen, and freeze-dried at −80 °C (±5 °C) and 0.029 mbar (Freezone; 4.5 L; LABCONCO, Fort Scott, KS, USA). The four freeze-dried samples, two from each location per year, were mixed and milled using IKA Multidrive Basic (BS000; Barcelona, Spain). Then it was passed through a 0.150 mm mesh to obtain a fine solid particle with a size under 0.037 mm obtaining a homogeneous and representative powdered sample, which presented a moisture content of 6.28 ± 0.38 g/100 g. The powdered samples were stored in the absence of light at −20 °C until analysis.

**Figure 1:**
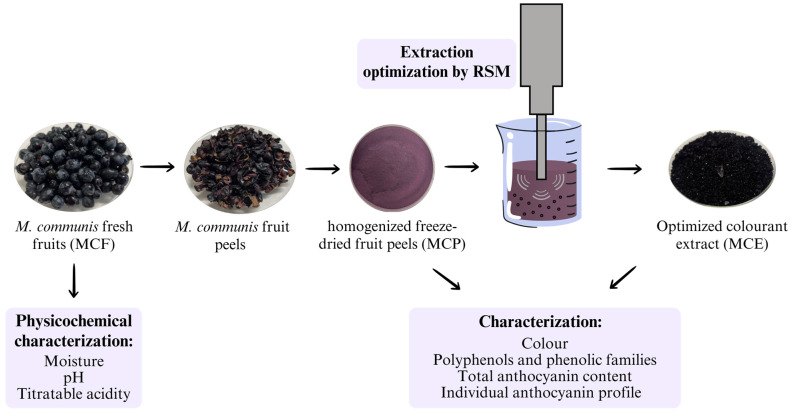
Samples of *Myrtus communis* used in the present study.

The different analytical methods were carried out in the following *Myrtus communis* samples (Figure 1):

(1) Fresh fruit sample (MCF, *M. communis* whole fruit) for initial physicochemical characterization was analyzed in the
(2) *M. communis* freeze-dried fruit peels (MCP), as a pool sample, for the determination of the optimal extraction conditions and developing an optimized colourant extract.
(3) *M. communis* freeze-dried fruit peels (MCP) and in the optimized colourant extract (MCE, *M. communis* extract) for the determination of bioactive compounds, namely anthocyanins characterization, total polyphenols and phenolic families.

### 2.2. Determination of the Optimal Extraction Conditions Through RSM

#### 2.2.1. Experimental Design

Based on preliminary studies, the factors selected for the evaluation of their combined influence on the anthocyanin extraction, and therefore as independent variables, were: time, evaluated at five levels (2.5–20 min); ultrasound power evaluated at 3 levels (250–500 W); solid–liquid ratio (S/L) evaluated at two levels (16.33–33.33 g/mL); and extraction solvent pH, also evaluated at 2 levels (3.0–6.0), generating a total of 60 possible combinations, each of which was carried out in duplicate, resulting in a total of 120 assays (Table 2).

#### 2.2.2. Ultrasound-Assisted Extraction Process

The ultrasound extraction process was carried out in the *M. communis* peel sample (MCP), which corresponds to the combined freeze-dried fruit peel samples from the two different locations collected in two consecutive years.

For each of the ultrasound-assisted extraction tests carried out to determine the optimal conditions of anthocyanin extraction, the subsequent procedure was followed: to 0.250 g of powdered *M. communis* peel sample (MCP), the corresponding quantity of extraction solvent (S/L ratio from 16.66 to 33.33; ethanol/water 80:20 (*v*/*v*)) was added, adjusted to the corresponding pH value (solvent pH from 3 to 6), according to the experimental design (Table 2). It was mixed and submitted to ultrasound with a Sonic Dismembrator model 705 (Fisherbrand, Pittsburg, PA, USA) (ultrasound power and time according to the experimental design). Then, the sample was centrifugated at 3500 rpm for 5 min and the supernatant was collected. A representative amount of the supernatant was used for the determination of the total anthocyanin content (Section Determination of Total Monomeric Anthocyanin) and the colour parameters (Section Individual Anthocyanin Profile) as explained in the responses criteria. The rest of the supernatant was rotatory evaporated (Rotavapor R-114; BÜCHI) for the elimination of the ethanol, then the aqueous extract was frozen at −20 °C and freeze-dried at −80 °C and 0.029 mbar (Freezone; 4.5 L; LABCONCO) for the obtention of a solid extract (optimized colourant extract, MCE).

#### 2.2.3. Responses Criteria Used to Evaluate the Extraction Process

Total anthocyanin content and the colour parameters saturation index (C*) and hue (h) were chosen as the response criteria for the evaluation and determination of the optimal condition of extraction of the anthocyanins from *M. communis*, which were carried out according to Section Determination of Total Monomeric Anthocyanin and Section 2.3.2, respectively.

#### 2.2.4. Mathematical Model Through Response Surface Methodology (RSM)

Response Surface Modelling (RSM) was used to analyze the combined effect of different experimental variables on the response of anthocyanin content. The independent factors considered were pH (X_1_), ultrasound power used (X_2_), time (X_3_), and solid/liquid ratio (X_4_). The RSM was constructed by fitting the experimental data to the following second-order polynomial equation:

(1) $$Y=b_{0}+\sum_{i=1}^{n}b_{i}X_{i}+\sum_{\begin{array}{c} i=1\\ j>1 \end{array}}^{n-1}\sum_{j=2}^{n}b_{ij}X_{i}X_{j}+\sum_{i=1}^{n}b_{ii}X_{i}^{2}$$

where Y denotes the response variable, namely total anthocyanin content. The coefficients b_0_, b_i_, b_ij_, and b_ii_ represent the constant, linear, interaction, and quadratic effects, respectively. Here, n stands for the total number of variables, which is 4 in this case. The NonlinearModelFit function from Mathematica (Version 11.1.1.0, Wolfram Research Inc., Champaign, IL, USA) was used to find the value of the RSM coefficients. The coefficient of determination (R^2^) and adjusted R^2^ returned by the NonlinearModelFit function were used to assess the accuracy of the model.

The fitted model was further used to find the experimental conditions of extraction, resulting in an optimal value of anthocyanin content. The FindMaximum function (also from Mathematica) was used with default parameters constraining the potential values of the experimental variables within the ranges defined by the minimum and maximum values observed in the experiments: 3 ≤ X_1_ ≤ 6, 50 ≤ X_2_ ≤ 100, 2.5 ≤ X_3_ ≤ 20 and 16.6 ≤ X_4_ ≤ 33.3 (as observed in Table 2).

### 2.3. Analytical Determinations

#### 2.3.1. Physical–Chemical Analysis

The fresh whole fruits of *M. communis* (MCF) were subjected to a physical–chemical analysis in terms of moisture, pH, and titratable acidity. Moisture was determined according to the AOAC 984.25 method [33]. pH was determined according to the AOAC 981.12 method [33] with a pH-metre Basic 20+, Crison. The titratable acidity was carried out through acid-base volumetry according to the AOAC 942.15 method [33]. °Brix was measured by refractometry at 20 °C (932.14C, A.O.A.C., 2006) in an Atago refractometer. All determinations were carried out in triplicate.

#### 2.3.2. Colour Characterization: CIELAB Parameter

Colour parameters were analyzed in the *M. communis* peel sample (MCP) and in the optimized colourant extract (MCE), as well as in each of the 120 assays carried out for the extraction optimization. CIELAB parameters were measured by the tristimulus colorimetry method [34]. For the freeze-dried peel sample and its optimized extract, the powdered sample was placed in a cylindrical cuvette of 5 × 1.3 cm up to full coverage of the base. For the measurement of the extraction optimization, 15 mL of each assay was placed in the cuvette. In both cases, the cuvette was placed in a colorimeter colorflex, Hunterlab under the following parameters: CIE L*a*b* colour space, illumination C, 10° and 45/0° geometry. Saturation index (C*) and hue (h) were determined from parameters a* and b* according to the following equations:

C*_ab_ = (a*^2^ + b*^2^)^1/2^ (2)

H_ab_ = arctan (b*/a*) (3)

#### 2.3.3. Anthocyanin Characterization: Total Monomeric Anthocyanin and Individual Anthocyanin Profile

##### Determination of Total Monomeric Anthocyanin

The total anthocyanin content (TAC) was determined in MCP and MCE, as well as in each of the 120 assays carried out for the extraction optimization. TAC was carried out by the pH differential method [35] with some modifications. For the determination of the optimal extraction, 100 µL of each assay was used; in the case of the *M. communis* peel sample (MCP) and optimized colourant extract (MCE), the determination was made through QUENCHER methodology [36]. Therefore, 10 ± 0.1 mg of each sample was used. Briefly, 10 mL of either KCl (Panreac, Barcelona, Spain) buffer (pH 1) or CH_3_CO_2_Na (Panreac, Barcelona, Spain) buffer (pH 4.5) was added to the sample, homogenized by vortexing, and after 15 min of orbital shaking, it was centrifugated for 5 min at 7000 rpm and filtered. Finally, the absorbance of the supernatant was measured at 510 and 700 nm in a UV–Vis microplate reader (Synergy HTX, Biotek; Santa Clara, CA, USA). A calibration curve of cyanidin-3-*O*-glucoside (3.125 to 500 µg/mL) was obtained; thus, the results were expressed as milligrams of cyanidin-3-glucoside (Sigma-Aldrich, Schnelldorf, Germany) per grams of sample (mg cya-3-glu/g, dw).

##### Individual Anthocyanin Profile

The individual anthocyanin profile was identified and quantified in MCP and MCE. For the individual anthocyanin profile determination and quantification, the extract was dissolved in water (10 mg/mL) and filtered through a PVDF filter disc of 0.22 µm. For the identification of the individual anthocyanins, the dissolved extract was injected into an HPLC equipment (Dionex Ultimate 3000 UPLC, Thermo Scientific, San Jose, CA, USA) coupled to a diode array detector (280, 330, 370, and 520 nm wavelengths) and an electrospray ionization mass spectrometer (Linear Ion Trap LTQ XL, Thermo Scientific) working in positive mode, utilizing an AQUA^®^ reverse phase C18 column (5 µm, 150 mm × 4.6 mm, Phenomenex; Alcobendas, Spain) at 35 °C for compound separation, following the gradient previously described by Gonçalves et al. [37]. The anthocyanin identification was carried out by comparison of the retention time, UV-Vis, and mass spectra with authentic standards and the literature data.

For the individual anthocyanins’ quantification, the dissolved extract was injected into UHPLC equipment (series 1290 Infinity II, Agilent Technologies, CA, USA) coupled to a diode array detector (DAD, series 1260 Infinity II, Agilent Technologies, CA, USA), as described by Vega et al. [38]. For compound separation, a Poroshell 120 SB-C18 column (4.6 mm × 75 mm; 2.7 µm; Agilent InfinityLab, Madrid, Spain) was used at 35 °C following the gradient previously described [38]. The quantification was carried out with ten level calibration curves obtained from pure standards (delphinidin-3-*O*-glucoside: y = 1042.533x + 9.285, cyanidin-3-*O*-glucoside: y = 1228.550x + 2.808, petunidin-3-*O*-glucoside: y = 821.793x + 2.358, pelargonidin-3-*O*-glucoside: y = 1362.533x + 7.749 and malvidin-3-*O*-glucoside: y = 1009.467x + 6.306) (Sigma-Aldrich, Schnelldorf, Germany). The results were expressed as milligrams per gram of sample (mg/g, dw).

#### 2.3.4. Determination of Total Polyphenols by QUENCHER Methodology

The total polyphenols (TP) were determined in MCP and MCE through the Fast Blue BB methodology [39,40] with some modifications. In brief, 1 ± 0.5 mg of the sample, 0.4 mL of 0.1% Fast Blue BB (Sigma-Aldrich, QuentinFallavier, France), 0.4 mL of 5% NaOH (Panreac, Barcelona, Spain), and 4 mL of distilled water were added, homogenizing by vortex after each reagent addition. After 45 min of orbital shaking the sample was centrifugated at 6500 rpm for 10 min, filtered, and the absorbance was measured at 420 nm in a UV–Vis microplate reader (Synergy HTX, Biotek; Santa Clara, CA, USA). A calibration curve of gallic acid (0.625 to 160 µg/mL) was obtained; therefore, the results were expressed as milligrams of gallic acid (Sigma-Aldrich, QuentinFallavier, France) equivalent per gram of sample (mg GAE/g dw).

##### Determination of Hydroxybenzoic Acids by QUENCHER Methodology

The hydroxybenzoic acids (HBC) were determined in MCP and MCE according to Bonoli et al. [41] with some modifications. In brief, 1 ± 0.5 mg of the sample, 0.5 mL of distilled water and 4 mL of 3% formic acid (Scharlau, Sentmenat, Spain) were added, and it was homogenized by vortexing. After 15 min of orbital shaking, it was centrifugated at 6500 rpm for 5 min, filtered, and the absorbance of the supernatant was measured at 280 nm in quartz cuvettes using a UV–Vis microplate reader (Synergy HTX, Biotek; Santa Clara, CA, USA). A calibration curve of gallic acid (3.125 to 400 µg/mL) was obtained, and then the results were expressed as milligrams of gallic acid equivalent per gram of sample (mg GAE/g dw).

##### Determination of Hydroxycinnamic Acids by QUENCHER Methodology

The hydroxycinnamic acids (HCC) were determined in MCP and MCE according to Bonoli et al. [41] with some modifications. Briefly, 1 ± 0.5 mg of the sample, 0.5 mL of distilled water, and 4 mL of methanol were added; it was homogenized by vortexing, and after 15 min of orbital shaking it was centrifugated at 6500 rpm for 5 min. Finally, the supernatant absorbance was measured at 320 nm in a UV–Vis microplate reader, (Synergy HTX, Biotek; Santa Clara, CA, USA). A calibration curve of ferulic acid (3.125 to 200 µg/mL) was obtained; hence, the results were expressed as milligrams of ferulic acid equivalent per gram of sample (mg FAE/g dw).

##### Flavonols by QUENCHER Methodology

Flavonols (FC) were determined in MCP and MCE according to Bonoli et al. [41] with some modifications. Briefly, 0.5 mL of distilled water and 4 mL of methanol were added to 1 ± 0.5 mg of the sample; it was homogenized by vortexing, and after 15 min of orbital shaking it was centrifugated at 6500 rpm and filtered. Lastly, the absorbance of the supernatant was measured at 370 nm with an UV–Vis microplate reader, (Synergy HTX, Biotek; Santa Clara, CA, USA). A calibration curve of quercetin (3.125 to 250 µg/mL) was obtained; hence, the results were expressed in milligrams of quercetin (Scharlau, Sentmenat, Spain) equivalent per gram of sample (mg QE/g, dw).

### 2.4. Statistical Analysis

All the analyses were carried out in triplicate and the results are presented as means values ± standard deviation. Student’s *t*-test was used to compare and identify the differences among the sets of means. All the analyses were performed using the XLSTAT programme (Addinsoft, 2024, New York, NY, USA).

## 3. Results and Discussion

### 3.1. Physicochemical Characterization of M. communis Fresh Fruits

As a first approach, the physicochemical properties and the colour parameters of *M. communis* fresh fruits (MCF) were determined in terms of moisture, pH, titratable acidity, Brix degrees, and colour parameters. *M. communis* fruits presented a moisture of 71.39 g/100 g of fruit, which is in accordance with previous data reported, which range from 61.7 to 75.7 g/100 g. In the same way, the obtained pH of 5.26 is in the range previously reported (4.37–6.56) [17,42]. Moreover, 58.47 mL of NaOH was needed for the neutralization of the acids present in 100 g of the fruits. ºBrix of 7.17 was obtained, evidencing an optimal maturity stage [17,42,43,44]. Regarding the colour parameters, measured by the CIEL*a*b* system, a low luminosity (L*: 34.14) was obtained, with an a* parameter of 9.97, indicating tones of red, and a b* parameter of −1.24, indicating tones of blue, evidencing a strong purple colour with a C* of 10.29 and a h of −0.11.

### 3.2. Determination of the Optimal Extraction Conditions Through RSM

The efficiency of anthocyanin extraction can be affected by several independent factors, including the nature of the anthocyanins; therefore, it becomes necessary to optimize the extraction process for each fruit and to evaluate each parameter independently. Therefore, the experimental domain of four factors was evaluated, meaning, that each factor was studied at different levels while maintaining the other factors constant. Specifically, pH and S/L ratio were evaluated in two factors: 3 and 6, and 16.66 and 33.33, respectively; the power of ultrasound in a three-level range, 250, 400, and 500 W; and time in a five-level range of 2.5, 5, 10, 15, and 20 min. Therefore, a total of 60 tests were obtained due to the different combinations of variables; moreover, each test was carried out in duplicate for a total of 120 assays. For each of these, the total monomeric anthocyanin content as well as the colour characteristics were determined (Table 3).

The response variables demonstrated notable variability, especially the colour parameters. More specifically, total anthocyanin content values ranged from 852.77 to 1106.55 mg cya-3-glu/g, while L* parameter values ranged from 5.24 to 23.01, a* parameter values from 17.27 to 40.11, and b* parameter values from 1.58 to 14.34. This extensive colour variability was also evident in chroma (17.37 to 42.13) and hue values (5.03 to 22.40). Given the wide range of colour parameters obtained and their sensitivity to external factors like pH, the extraction conditions were optimized with a focus on maximizing anthocyanin yield; therefore, total anthocyanin content was chosen as the primary criterion.

The results from each experiment (Table 3) were used to build a Response Surface Model (RSM) to extend the findings beyond the selected conditions and to gain a deeper understanding of the factors affecting anthocyanin concentration. For this the coefficients of the second-order polynomial equation (Equation (4)) were calculated resulting in the following model:

(4) $$Y=603.765+49.9719X_{1}+2.78618X_{2}-8.49634X_{3}+8.3743X_{4}-1.0871X_{1}^{2}-0.000574667X_{2}^{2}+0.110137X_{3}^{2}-0.0681449X_{4}^{2}\;-0.200884X_{1}X_{2}+1.29339X_{1}X_{3}-0.445722X_{1}X_{4}-0.0105695X_{2}X_{3}-0.0387428X_{2}X_{4}+0.0427951X_{3}X_{4}$$

where X_1_ refers to the pH, X_2_ to the ultrasound power used, X_3_ to time, X_4_ to the solid/liquid ratio, and Y represents the anthocyanin content. The values of the coefficient of determination (R^2^) and adjusted R^2^ were 0.998475 and 0.997966, respectively, demonstrating an excellent fit of the model.

**Figure 2:**
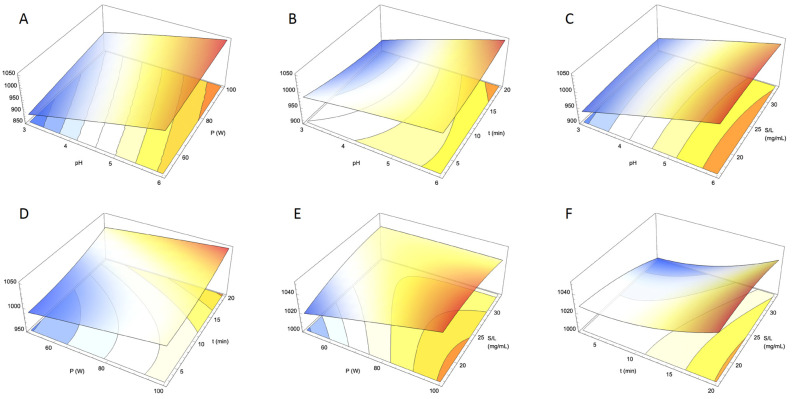
Response surface graphs of the combined effect of the independent variable (pH, ultrasound power used (P), extraction time (t) and solid/liquid ratio (S/L)) on total anthocyanin content (in mg cya-3-glu/g; Y-axis). Excluded variables in each graph were positioned at their optimal values. (**A**): combined effect of pH (X-axis) and P (Z-axis); (**B**): combined effect of pH (X-axis) and t (Z-axis); (**C**): combined effect of pH (X-axis) and S/L (Z-axis); (**D**): combined effect of P (X-axis) and t (Z-axis); (**E**): combined effect of P (X-axis) and S/L (Z-axis); (**F**): combined effect of t (X-axis) and S/L (Z-axis).

In this modelling approach, the magnitude of the coefficients indicates the strength and type of relationship each variable has with the response variable (Y). Variables with larger absolute coefficients have a more significant impact on the results, considering the magnitudes of the variables. In the developed model, pH (X_1_) and its linear term exhibit the most substantial effect on the response variable, followed by the linear terms of time (X_3_) and the solid/liquid ratio (X_4_). Interestingly, the interaction between time and pH (X_1_ X_3_) presents a higher value after the linear terms. Contrary to the expected, the anthocyanin content increases with higher pH levels (Figure 2). For the time variable, although the linear term has a negative effect, this is offset by the quadratic and interaction terms, resulting in an overall increase in anthocyanin content. A similar but less pronounced effect is observed for the solid/liquid ratio.

Using the developed response surface method, the optimal experimental conditions to achieve the highest anthocyanin content were identified within the established experimental range. According to the optimization procedure, the maximum anthocyanin content of 10.51 mg cya-3-glu/g was achieved at pH 6, with an ultrasound power of 100 W, an extraction time of 20 min, and a solid/liquid ratio of 19.6756 mg/mL.

### 3.3. Anthocyanin Characterization in M. communis Peel Sample (MCP) and Its Optimized Colourant Extract (MCE)

The total monomeric anthocyanin (TAC) was determined in MCP and MCE, obtaining contents of 25.53 mg cya-3-glu/g (dw) and 47.51 ± 1.50 mg cya-3-glu/g (dw), respectively. The TAC values of *M. communis* fruit samples were remarkably superior to those previously reported for two different cultivars harvested in two different years in Italy, which presented a range from 0.029 to 10.11 mg/g (dw) [18]. Moreover, the TAC values of the optimized extract surpassed previous reports, with values ranging from 0.13 to 8.99 mg/g (dw) in extracts of fruits collected in Italy [45]. In addition, the anthocyanin recovery yield was notably enhanced compared to previously reported extraction methods. For instance, Bouaoudia-Madi et al. [31] compared conventional solvent extraction with microwave-assisted extraction, evidencing a 1.3-fold increase in the anthocyanin content. Similarly, González de Peredo et al. [46] proposed an ultrasound-assisted extraction, yielding slightly better recovery rates than the Bouaoudia-Madi et al. method. In addition, the remarkably high anthocyanin content in MCE can be evidenced when compared to extracts from other matrices known for their high anthocyanin levels. For example, an optimized extract from wild blackberry fruits (*Rubus fruticosus* L.) presented a TAC of 11.2 mg cya-3-glu/g (dw), which is lower than the value observed in MCE [47].

**Figure 3:**
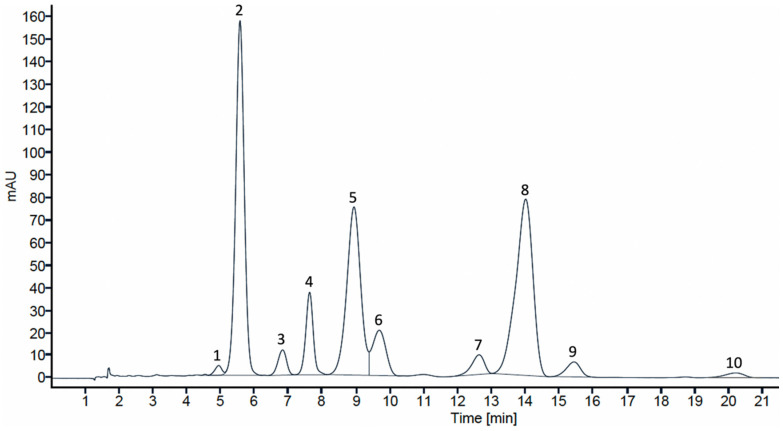
Chromatographic profile at 520 nm of the anthocyanin compounds in *Myrtus communis* optimized colourant extract (1: delphinidin-*O*-diglucoside; 2: delphinidin-3-*O*-glucoside 3: cyanidin-3-*O*-galactoside; 4: cyanidin-3-*O*-glucoside 5: petunidin-3-*O*-glucoside; 6: Malvidin-*O*-dihexoside; 7: pelargonidin derivative; 8: malvidin-3-*O*-glucoside; 9: petunidin-3-*O*-arabinoside; 10: malvidin-3-*O*-arabinoside).

The individual anthocyanin profile was identified in MCE, evidencing a chromatographic profile with the presence of 10 anthocyanins (Figure 3). Moreover, Table 4 contains the tentative identification and quantification of anthocyanin compounds in the fruit sample and in the optimized colourant extract. Peaks 2 (delphinidin-3-*O-*glucoside), 4 (cyanidin-3-*O*-glucoside), 5 (petunidin-3-*O*-glucoside), and 8 (malvidin-3-*O*-glucoside) were identified by comparison with authentic standards. These compounds showed [M^+^] at m/z 465, 449, 479, and 493, as well as a unique MS2 fragment of 303, 287, 317, and 331, respectively. In all cases, neutral losses of 162 Da, corresponding to the loss of a hexose unit were observed, confirming the identification made with authentic standards.

The other peaks were identified by comparing the compounds’ fragmentation patterns with the available literature data. Peak 1 showed an [M]^+^ ion at *m/z* 627 and a fragment ion at *m*/*z* 303, indicating a delphinidin aglycone after the loss of two hexose units ([M-324]^+^). Based on this evidence and prior identification of anthocyanins of this fruit, peak 1 was tentatively identified as delphinidin-*O*-diglucoside [19,32].

Peak 3 exhibited an [M]^+^ ion at *m*/*z* 449, yielding a fragment ion at *m*/*z* 287, which is consistent with cyanidin after the loss of a hexose unit (162 Da). As peak 4 has been unequivocally identified as cyanidin-3-*O*-glucoside, the sugar moiety in compound 3 was designated as galactoside, another common hexose found in foods [48]. Previous studies have reported that galactose-linked compounds elute earlier than glucose-linked ones [48]. Consequently, peak 3 was assigned as cyanidin-3-*O*-galactoside [19,32].

Peak 6 ([M]^+^ at *m*/*z* 655) showed a fragment ion at *m*/*z* 331 in its MS² spectrum, characteristic of malvidin after the neutral loss of two hexose units ([M-324]^+^). Thus, it was identified as malvidin-*O*-dihexoside. Peak 7 ([M]^+^ at *m*/*z* 463) exhibited a fragment ion at *m*/*z* 271 in the MS² spectrum, which is characteristic of pelargonidin, and was designated as a pelargonidin derivative.

Peaks 9 ([M]^+^ at *m*/*z* 449) and 10 ([M]^+^ at *m*/*z* 463) produced fragment ions at *m*/*z* 317 and *m*/*z* 331, respectively, suggesting the loss of a pentose group ([M-132]^+^). Based on these fragmentation patterns and prior identification of anthocyanins in *M. communis* fruits, peaks 9 and 10 were tentatively identified as petunidin-3-*O*-arabinoside and malvidin-3-*O*-arabinoside, respectively [32,49].

Overall, the anthocyanin variety in MCP was evident, including delphinidin, cyanidin, petunidin, pelargonidin, and malvidin derivatives, the latter being the most prevalent. Malvidin-3-glucoside was the predominant anthocyanin with 6.00 mg/g of fruit, closely followed by delphinidin-3-glucoside (5.48 mg/g of fruit) and petunidin-3-glucoside (5.48 mg/g of fruit), which agrees with what was previously reported by Messaoud et al. [49]. Nonetheless, the content of each anthocyanin found in this work was higher than previously reported by the same author. Additionally, MCE showed an increase in the quantity of each anthocyanin, evidencing the success of the extraction optimization process, with a total of identified and quantified anthocyanins of 35.39 mg/g of extract, which is 1.7 times higher than the total identified and quantified in MCP (20.62 mg/g of fruit).

### 3.4. Total Polyphenols and Phenolic Families in M. communis Peel Sample (MCP) and Its Optimized Colourant Extract (MCE)

MCP and MCE were characterized for total phenolic content and different phenolic families, as shown in Table 5. In general, the hydroethanolic extraction process carried out was highly efficient, producing an extract rich not only in anthocyanins but also in a wide range of other phenolic compounds.

A total of 190.23 mg GAE of phenolic content/g (dw) was found through Fast Blue BB methodology in MCP. This value was higher than previously reported by other authors from fruits collected in Tunisia or Turkey, whose content ranged from 5.31 to 63.2 mg GAE/g of fruit [17,20,43,49]. This observed difference can be attributed to several factors: firstly, the use of the Fast Blue BB method instead of the traditional Folin–Ciocalteu method, commonly employed for the determination of total polyphenols. This method can be prone to overestimation, as the Folin–Ciocalteu reagent can be reduced by other agents such as sugars or vitamins [50]. Secondly, the use of the QUENCHER methodology provides more reliable results, as it allows the solid sample to be in direct contact with the reagents, enabling the quantification of both soluble and insoluble compounds [40]. Finally, it is well-established that wild fruits collected from natural habitats present a wider variety and higher levels of bioactive compounds, as secondary metabolites serving as a protective response to environmental stress [51].

Among the phenolic families, hydroxybenzoic acids were most abundant with 34.99 mg GAE/g, followed by flavonols with a content of 10.61 mg QE/g, while hydroxycinnamic acids were the least abundant with a content of 9.35 mg FAE/g. These results align with those previously reported by other authors, such as Messaoud et al. [49], who reported values of flavonols of 3.5 mg CE/g, (dw) for fruits collected in Tunisia.

MCE presented a significative enhancement in the content of bioactive compounds compared to MCP, where all the families increased by 1.4 to 1.8 times. Moreover, MCE presented the same phenolic profile as MCP, evidencing a successful extraction of most bioactive compounds. The total phenolic content (TPC) exhibited a notable value of 271.18 mg GAE/g of optimized colourant extract, surpassing previously reported figures, such as those from fruits collected across seven different locations in France (21.84 to 67.43 mg/g of extract) [52]. Other authors have reported TPC values from 0.02 to 4.57 g GAE/L for aqueous and ethanolic extracts from fruits collected in Italy. Hydroxybenzoic acids (HBC) emerged as the predominant phenolic family, followed by flavonols content (FC), while hydroxycinnamic acids (HCC) were the least representative in MCE. Notably, FC values obtained were superior to those reported for different extracts from fruits gathered in seven locations in France, with values ranging between 5.19 and 15.21 mg/g (dw) [52].

The determination of several of these phenolic families has not been reported; therefore, there are no available data for direct comparison. However, the contents found in this work for MCP exceeded those reported for other fruits known for their high content of bioactive compounds, especially anthocyanins. For example, highbush blueberry (*Vaccinium corymbosum* L.) from four different cultivars presented values of TPC between 15 and 55 mg GAE/g (dw) and HCC between 3 and 6 mg/g (dw) [53]. Similarly, black chokeberry (*Aronia melanocarpa* (Michx.) *Elliott*) presented content of HCC between 1.16 and 2.31 mg/g (dw) and FC between 0.57 and 1.26 mg/g (dw) [54]. Blackcurrant (*Ribes nigrum* L., cultivars Ojebyn and Titan) presented values of HBC between 0.06 and 0.12 mg GAE/g (dw), HCC between 0.58 and 0.93 mg CAE/g (dw), and FC between 0.72 and 0.87 mg RE/g (dw) [55].

## 4. Conclusions

*M. communis* fruit peel sample (MCP), treated as a by-product, presented remarkable anthocyanin content and an intense dark purple colour. Using Response Surface Methodology (RSM), the optimal extraction conditions were determined as 20 min, pH 6, ultrasound potence of 500 W, and an S/L ratio of 19.68 g/L for maximizing anthocyanin extraction from MPC through ultrasound-assisted extraction.

The optimized colourant extract (MCE) obtained presented a polyphenol content of 271.18 mg GAE/g and a total anthocyanin content of 47.51 mg cya-3-glu/g, establishing anthocyanins as the predominant bioactive compound. This anthocyanin content represents a 1.86-fold increase compared to MCP, evidencing the effectiveness of the optimized extraction process. The analysis of the individual anthocyanin profile revealed a diverse range of compounds, including derivatives of delphinidin, cyanidin, petunidin, pelargonidin, and malvidin, with malvidin-3-*O*-glucoside being the most abundant (10.30 mg/g of extract).

In conclusion, this study presents an optimized ultrasound-assisted extraction method to obtain an extract rich in anthocyanins and other bioactive compounds from *M. communis* fruit peels. The optimized extract demonstrates strong potential as a natural food colourant, offering both vibrant purple coloration and a high concentration of anthocyanins and phenolic compounds

## Figures and Tables

**Table 1:** *Myrtus communis* L. fruits and their collection sites and coordinates.

	Location 1	Location 2
	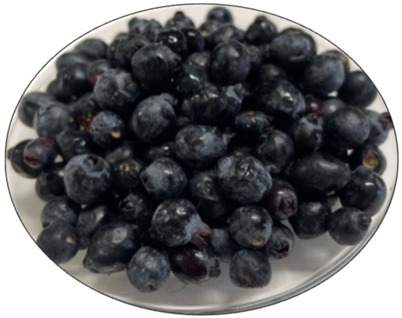	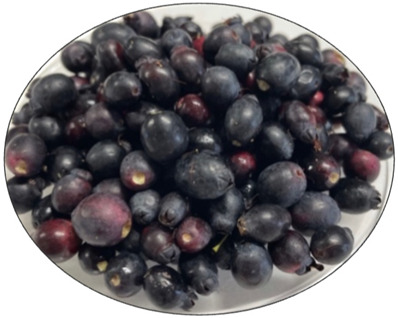
Location	Parque Natural de l’Albufera, El Palmar (Spain)	Paratge Natural Municipal, Sierra de la Murta, Alcira (Spain)
Latitude	39°20′18.6″ N	39°07′43.6″ N
Longitude	0°19′09.1″ W	0°21′19.7″ W
Years of collection	2021, 2022	2021, 2022

**Table 2:** Experimental design for the optimization of the anthocyanin extraction through ultrasound assisted extraction in MCP sample.

Code	pH	P (W)	t (min)	S/L (mg/mL)	Code	pH	P (W)	t (min)	S/L (mg/mL)
1	3	250	2.5	33.33	31	6	250	2.5	33.33
2	3	250	5	33.33	32	6	250	5	33.33
3	3	250	10	33.33	33	6	250	10	33.33
4	3	250	15	33.33	34	6	250	15	33.33
5	3	250	20	33.33	35	6	250	20	33.33
6	3	400	2.5	33.33	36	6	400	2.5	33.33
7	3	400	5	33.33	37	6	400	5	33.33
8	3	400	10	33.33	38	6	400	10	33.33
9	3	400	15	33.33	39	6	400	15	33.33
10	3	400	20	33.33	40	6	400	20	33.33
11	3	500	2.5	33.33	41	6	500	2.5	33.33
12	3	500	5	33.33	42	6	500	5	33.33
13	3	500	10	33.33	43	6	500	10	33.33
14	3	500	15	33.33	44	6	500	15	33.33
15	3	500	20	33.33	45	6	500	20	33.33
16	3	250	2.5	16.66	46	6	250	2.5	16.66
17	3	250	5	16.66	47	6	250	5	16.66
18	3	250	10	16.66	48	6	250	10	16.66
19	3	250	15	16.66	49	6	250	15	16.66
20	3	250	20	16.66	50	6	250	20	16.66
21	3	400	2.5	16.66	51	6	400	2.5	16.66
22	3	400	5	16.66	52	6	400	5	16.66
23	3	400	10	16.66	53	6	400	10	16.66
24	3	400	15	16.66	54	6	400	15	16.66
25	3	400	20	16.66	55	6	400	20	16.66
26	3	500	2.5	16.66	56	6	500	2.5	16.66
27	3	500	5	16.66	57	6	500	5	16.66
28	3	500	10	16.66	58	6	500	10	16.66
29	3	500	15	16.66	59	6	500	15	16.66
30	3	500	20	16.66	60	6	500	20	16.66

**Table 3:** Independent variable used in each test of the experimental design and the results obtained in each response criteria evaluated.

	Independent Variables Evaluated						
Code	TAC (mg cya-3-glu/g)	L*	a*	b*	C*	h	RGB
1	9.54 ± 0.00	11.46 ± 0.08	36.96 ± 1.92	13.25 ± 0.21	38.93 ± 1.87	19.84 ± 0.76	
2	9.33 ± 0.01	12.81 ± 0.41	38.58 ± 0.38	13.24 ± 0.29	40.89 ± 0.38	18.94 ± 0.35	
3	9.21 ± 0.51	13.67 ± 0.37	39.14 ± 0.58	13.29 ± 0.46	41.50 ± 0.54	18.87 ± 0.58	
4	8.55 ± 0.03	14.02 ± 0.56	39.26 ± 0.85	13.01 ± 0.60	41.22 ± 0.60	18.50 ± 1.20	
5	9.21 ± 0.08	12.84 ± 0.45	38.67 ± 0.36	13.37 ± 0.13	40.99 ± 0.30	18.99 ± 0.22	
6	9.50 ± 0.19	13.30 ± 0.12	39.29 ± 0.35	13.46 ± 0.77	41.45 ± 0.57	18.68 ± 0.80	
7	9.36 ± 0.23	12.74 ± 0.70	38.16 ± 0.61	13.31 ± 0.39	40.32 ± 0.74	19.24 ± 0.28	
8	9.36 ± 0.08	13.17 ± 0.14	38.52 ± 0.59	13.47 ± 0.62	40.66 ± 0.72	19.20 ± 0.66	
9	9.48 ± 0.81	12.80 ± 0.47	38.26 ± 0.21	13.35 ± 0.52	40.46 ± 0.28	19.02 ± 0.50	
10	9.58 ± 0.45	13.14 ± 0.59	38.40 ± 0.05	13.03 ± 0.49	40.55 ± 0.18	18.65 ± 0.69	
11	10.36 ± 0.22	12.61 ± 0.41	37.56 ± 0.12	13.52 ± 0.19	39.98 ± 0.07	19.81 ± 0.27	
12	10.10 ± 0.05	13.28 ± 0.12	38.66 ± 0.56	13.22 ± 0.72	41.04 ± 0.69	18.96 ± 0.78	
13	9.18 ± 0.30	13.39 ± 0.30	38.22 ± 1.12	12.96 ± 0.77	40.57 ± 1.31	18.76 ± 0.65	
14	9.00 ± 0.75	13.00 ± 0.15	37.48 ± 0.24	12.55 ± 0.36	39.55 ± 0.33	18.63 ± 0.37	
15	9.37 ± 0.57	12.56 ± 0.84	38.48 ± 1.30	14.13 ± 0.16	40.80 ± 1.23	20.34 ± 0.71	
16	9.09 ± 0.08	6.87 ± 0.56	29.71 ± 0.24	8.33 ± 0.15	30.90 ± 0.26	15.69 ± 0.22	
17	8.69 ± 0.40	6.50 ± 0.12	29.77 ± 0.43	8.30 ± 0.22	31.06 ± 0.31	15.61 ± 0.26	
18	9.21 ± 0.66	6.31 ± 0.02	28.45 ± 0.30	7.42 ± 0.34	29.39 ± 0.28	14.89 ± 0.42	
19	8.89 ± 0.00	6.00 ± 0.40	28.93 ± 0.62	7.67 ± 0.34	29.83 ± 0.71	14.77 ± 0.35	
20	8.53 ± 0.35	6.39 ± 0.17	29.33 ± 0.40	7.99 ± 0.25	30.54 ± 0.26	15.30 ± 0.47	
21	10.09 ± 1.29	6.65 ± 0.38	28.05 ± 0.26	7.49 ± 0.25	29.03 ± 0.28	14.95 ± 0.58	
22	9.45 ± 0.07	6.09 ± 0.18	27.84 ± 0.40	7.22 ± 0.29	28.83 ± 0.45	14.61 ± 0.46	
23	9.30 ± 0.36	5.82 ± 0.38	28.70 ± 0.48	7.36 ± 0.22	29.54 ± 0.52	14.37 ± 0.29	
24	8.77 ± 0.28	6.04 ± 0.20	27.85 ± 1.24	6.89 ± 0.35	28.47 ± 1.30	13.88 ± 0.33	
25	9.39 ± 0.04	6.79 ± 0.21	28.31 ± 1.05	7.11 ± 0.60	29.37 ± 1.20	14.25 ± 0.61	
26	8.97 ± 0.16	6.14 ± 0.28	28.02 ± 1.03	6.82 ± 0.26	28.70 ± 1.24	13.44 ± 0.93	
27	9.77 ± 0.35	6.59 ± 0.15	27.37 ± 0.98	6.61 ± 0.53	27.99 ± 1.09	13.65 ± 0.72	
28	9.29 ± 0.06	6.06 ± 0.49	28.50 ± 0.25	7.07 ± 0.11	29.34 ± 0.24	13.95 ± 0.33	
29	9.00 ± 0.14	6.43 ± 0.36	27.65 ± 0.98	6.10 ± 0.08	28.16 ± 1.16	12.25 ± 0.36	
30	10.15 ± 0.62	5.94 ± 0.40	27.27 ± 0.96	6.41 ± 0.57	28.25 ± 0.99	13.23 ± 0.86	
31	9.14 ± 0.40	22.66 ± 0.21	18.13 ± 0.34	2.14 ± 0.17	18.21 ± 0.38	6.61 ± 0.34	
32	9.39 ± 0.31	20.26 ± 0.46	18.35 ± 0.13	2.13 ± 0.09	18.45 ± 0.15	6.57 ± 0.23	
33	10.44 ± 0.39	22.58 ± 0.36	17.60 ± 0.29	1.73 ± 0.15	17.64 ± 0.28	5.73 ± 0.28	
34	11.07 ± 0.65	21.49 ± 0.52	17.90 ± 0.25	2.75 ± 0.11	18.08 ± 0.28	8.80 ± 0.33	
35	10.63 ± 0.22	21.02 ± 0.28	17.99 ± 0.34	2.33 ± 0.18	18.11 ± 0.35	7.53 ± 0.64	
36	10.02 ± 0.07	21.02 ± 0.62	18.31 ± 0.32	1.94 ± 0.08	18.38 ± 0.37	5.91 ± 0.26	
37	10.65 ± 0.54	21.58 ± 0.35	18.20 ± 0.32	3.37 ± 0.21	18.53 ± 0.31	10.36 ± 0.81	
38	9.96 ± 0.06	19.94 ± 0.88	18.23 ± 0.32	4.59 ± 0.42	18.86 ± 0.20	13.90 ± 1.28	
39	10.06 ± 0.60	20.25 ± 0.47	18.54 ± 0.12	2.34 ± 0.10	18.67 ± 0.12	7.19 ± 0.36	
40	10.16 ± 0.24	20.86 ± 0.75	18.28 ± 0.22	4.62 ± 0.23	19.27 ± 0.79	15.47 ± 0.64	
41	10.02 ± 0.19	20.16 ± 0.28	18.12 ± 0.24	5.42 ± 0.03	18.98 ± 0.04	17.13 ± 0.11	
42	9.76 ± 0.67	19.50 ± 0.18	18.84 ± 0.14	3.36 ± 0.06	19.14 ± 0.22	9.80 ± 0.18	
43	10.20 ± 0.01	20.81 ± 0.22	18.03 ± 0.23	4.95 ± 0.20	18.66 ± 0.10	15.75 ± 1.61	
44	10.01 ± 0.01	20.34 ± 0.05	18.41 ± 0.69	4.52 ± 0.08	19.35 ± 1.30	14.78 ± 0.23	
45	10.28 ± 0.04	21.15 ± 1.04	18.18 ± 0.25	2.69 ± 0.07	18.34 ± 0.26	8.43 ± 0.18	
46	10.70 ± 0.20	7.12 ± 0.59	22.14 ± 0.31	3.35 ± 0.24	22.47 ± 0.19	8.38 ± 0.75	
47	9.72 ± 0.59	8.45 ± 0.08	21.92 ± 0.57	3.48 ± 0.05	22.08 ± 0.45	9.44 ± 0.20	
48	9.83 ± 0.60	8.01 ± 0.07	22.50 ± 0.43	3.26 ± 0.16	22.80 ± 0.44	8.29 ± 0.51	
49	9.66 ± 1.39	7.14 ± 0.17	22.21 ± 0.72	4.65 ± 0.42	22.86 ± 0.75	11.94 ± 0.68	
50	10.02 ± 0.52	7.70 ± 0.33	22.27 ± 0.14	3.85 ± 0.14	22.58 ± 0.16	9.73 ± 0.28	
51	9.79 ± 0.09	7.12 ± 0.30	21.98 ± 0.51	3.16 ± 0.26	22.31 ± 0.52	8.18 ± 0.61	
52	9.93 ± 0.67	7.25 ± 0.27	22.44 ± 0.48	4.13 ± 0.25	22.76 ± 0.55	10.32 ± 0.49	
53	9.99 ± 1.22	7.74 ± 0.19	21.57 ± 0.09	4.08 ± 0.28	21.93 ± 0.08	10.54 ± 0.68	
54	10.17 ± 0.04	7.45 ± 0.34	21.69 ± 0.40	3.83 ± 0.26	21.92 ± 0.24	10.23 ± 0.72	
55	10.13 ± 1.34	7.46 ± 0.35	21.61 ± 0.27	3.87 ± 0.15	21.92 ± 0.26	10.25 ± 0.47	
56	9.86 ± 0.31	7.46 ± 0.13	21.11 ± 0.56	3.69 ± 0.27	21.47 ± 0.66	9.97 ± 0.54	
57	10.50 ± 0.02	7.42 ± 0.18	21.47 ± 0.29	4.15 ± 0.19	21.95 ± 0.27	11.03 ± 0.37	
58	10.90 ± 0.34	7.42 ± 0.34	22.02 ± 0.47	4.33 ± 0.30	22.34 ± 0.50	10.97 ± 0.52	
59	11.04 ± 0.87	7.79 ± 0.21	21.82 ± 0.40	4.80 ± 0.29	22.26 ± 0.43	12.29 ± 0.61	
60	10.19 ± 0.70	7.28 ± 0.38	21.48 ± 0.81	3.98 ± 0.32	21.96 ± 0.91	10.45 ± 0.95	

TAC: total anthocyanin content; cya-3-glu: cyanidin-3-*O*-glucoside; L*: luminosity; a*: red–green; b*: yellow–blue; C*: chroma; h: hue. RGB: colour obtained with red-green-blue model.

**Table 4:** Individual anthocyanin identification and quantification of *Myrtus communis* peel sample (MCP) and optimized colourant extract (MCE).

Peak	Rt	UV	[M]^+^	MS^2^	Tentative Identification	Quantification (mg/g, dw)	
						*M. communis* Peel Sample (MCP)	*M. communis* Extract (MCE)
1 ^A^	4.91	523	627	303	Delphinidin-*O*-diglucoside	0.11 ± 0.00 ^b^	0.19 ± 0.00 ^a^
2 ^A^	5.53	523	465	303	Delphinidin-3-*O*-glucoside	5.48 ± 0.01 ^b^	9.41 ± 0.01 ^a^
3 ^B^	6.82	516	449	287	Cyanidin-3-*O*-galactoside	0.34 ± 0.00 ^b^	0.58 ± 0.00 ^a^
4 ^B^	7.62	516	449	287	Cyanidin-3-*O*-glucoside	0.95 ± 0.01 ^b^	1.63 ± 0.01 ^a^
5 ^C^	8.93	524	479	317	Petunidin-3-*O*-glucoside	5.48 ± 0.02 ^b^	9.41 ± 0.03 ^a^
6 ^D^	9.69	524	655	33	Malvidin-*O*-dihexoside	1.15 ± 0.00 ^b^	1.97 ± 0.01 ^a^
7 ^E^	12.65	517	463	271	Pelargonidin derivative	0.40 ± 0.00 ^b^	0.69 ± 0.00 ^a^
8 ^D^	14.01	526	493	331	Malvidin-3-*O*-glucoside	6.00 ± 0.01 ^b^	10.30 ± 0.01 ^a^
9 ^C^	15.47	526	449	317	Petunidin-3-*O*-arabinoside	0.53 ± 0.00 ^b^	0.90 ± 0.00 ^a^
10 ^D^	20.26	528	463	331	Malvidin-3-*O*-arabinoside	0.18 ± 0.00 ^b^	0.31 ± 0.01 ^a^

In the peak column, each capital superscript letter means de calibration curve used for quantification. A—delphinidin-3-*O*-glucoside (y = 1047.4x + 8.3518); B—cyanidin-3-*O*-glucoside (y = 1217.5x + 3.2479); C—petunidin-3-*O*-glucoside (y = 832.74x + 2.6594); D—malvidin-3-*O*glucoside (y = 1011x + 6.8629); E—pelargonidin-3-*O*-glucoside (y = 1371.9x + 7.8528). Different small superscript letters in each line mean statistically significant differences (*p* < 0.05) compared by Student’s *t*-test.

**Table 5:** Chemical composition and bioactive properties of *Myrtus communis* L. fruit and the optimized extract.

	*M. communis* Peel (MCP)	*M. communis* Extract (MCE)
Total phenolic (mg GAE/g, dw)	190.23 ± 15.13 ^b^	271.18 ± 22.89 ^a^
Hydroxybenzoic acids (mg GAE/g, dw)	34.99 ± 1.56 ^b^	44.96 ± 0.59 ^a^
Hydroxycinnamic acids (mg FAE/g, dw)	9.35 ± 0.39 ^b^	17.57 ± 1.52 ^a^
Flavonols (mg QE/g, dw)	10.61 ± 0.48 ^b^	20.33 ± 1.63 ^a^

GAE: Gallic acid equivalent; cya-3-glu: cyanidin-3-*O*-glucoside; FAE: ferulic acid equivalent, QE: quercetin equivalent. Different small superscript letters in each line mean statistically significant differences (*p* < 0.05) compared by Student’s *t*-test.

## Data Availability

The original contributions presented in this study are included in the article. Further inquiries can be directed to the corresponding author.

## References

[1] EFSA Food Colours. https://www.efsa.europa.eu/en/topics/topic/food-colours.

[2] Vega E.N., Ciudad-Mulero M., Fernández-Ruiz V., Barros L., Morales P. (2023). Natural Sources of Food Colorants as Potential Substitutes for Artificial Additives. Foods.

[3] Spence C. (2022). On the Manipulation, and Meaning(s), of Color in Food: A Historical Perspective. J. Food Sci..

[4] Feingold B.F. (1976). Hyperkinesis and Learning Disabilities Linked to the Lngestion of Artificial Food Colors and Flavors. J. Learn. Disabil..

[5] McCann D., Barrett A., Cooper A., Crumpler D., Dalen L., Grimshaw K., Kitchin E., Lok K., Porteous L., Prince E. (2007). Food Additives and Hyperactive Behaviour in 3-Year-Old and 8/9-Year-Old Children in the Community: A Randomised, Double-Blinded, Placebo-Controlled Trial. Lancet.

[6] Amin K.A., Fawzia S.A.-S. (2018). Toxicological and Safety Assessment of Tartrazine as a Synthetic Food Additive on Health Biomarkers: A Review. Afr. J. Biotechnol..

[7] Pestana S., Moreira M., Olej B. (2010). Safety of Ingestion of Yellow Tartrazine by Double-Blind Placebo Controlled Challenge in 26 Atopic Adults. Allergol. Immunopathol..

[8] Cena H., Calder P.C. (2020). Defining a Healthy Diet: Evidence for the Role of Contemporary Dietary Patterns in Health and Disease. Nutrients.

[9] Khoo H.E., Azlan A., Tang S.T., Lim S.M. (2017). Anthocyanidins and Anthocyanins: Colored Pigments as Food, Pharmaceutical Ingredients, and the Potential Health Benefits. Food Nutr. Res..

[10] Zhang J., Celli G.B., Brooks M.S., Su-Ling M., Celli G.B. (2019). Natural Sources of Anthocyanins. Anthocyanins from Natural Sources: Exploiting Targeted Delivery for Improved Health.

[11] Andersen Ø.M., Jordheim M., Wallace T.C., Giusti M. (2010). Basic Anthocyanin Chemistry and Dietary Sources. Anthocyanins in Health and Disease.

[12] Morata A., López C., Tesfaye W., González C., Escott C., Grumezescu A.M., Holban A.M. (2019). Anthocyanins as Natural Pigments in Beverages. Value-Added Ingredients and Enrichments of Beverages.

[13] Câmara J.S., Locatelli M., Pereira J.A.M., Oliveira H., Arlorio M., Fernandes I., Perestrelo R., Freitas V., Bordiga M. (2022). Behind the Scenes of Anthocyanins—From the Health Benefits to Potential Applications in Food, Pharmaceutical and Cosmetic Fields. Nutrients.

[14] Tardío J., Macía M.J., Morales R. (2018). Myrtus communis L. Inventario Español de los Conocimientos Tradicionales relativos a la Biodiversidad. Fase II.

[15] Tardío J., Sánchez-Mata M., Morales R., Molina M., García-Herrera P., Morales P., Díez-Marqués C., Fernández-Ruiz V., Cámara M., Pardo de Santayana M., Sánchez-Mata M., Tardío J. (2016). Ethnobotanical and Food Composition Monographs of Selected Mediterranean Wild Edible Plants. Mediterranean Wild Edible Plants. Ethnobotany and Food Composition Tables.

[16] Usai M., Marchetti M., Culeddu N., Mulas M. (2018). Chemical Composition of Myrtle (*Myrtus communis* L.) Berries Essential Oils as Observed in a Collection of Genotypes. Molecules.

[17] Fernández-Ruiz V., Morales P., Ruiz-Rodríguez B.M., Isasa E.T., Ferreira C.F.R., Morales P., Barros L. (2016). Nutrients and Bioactive Compounds in Wild Fruits Through Different Continents. Wild Plants, Mushrooms and Nuts.

[18] Fadda A., Mulas M. (2010). Chemical Changes during Myrtle (*Myrtus communis* L.) Fruit Development and Ripening. Sci. Hortic..

[19] Maldini M., Chessa M., Petretto G.L., Montoro P., Rourke J.P., Foddai M., Nicoletti M., Pintore G. (2016). Profiling and Simultaneous Quantitative Determination of Anthocyanins in Wild *Myrtus communis* L. Berries from Different Geographical Areas in Sardinia and Their Comparative Evaluation. Phytochem. Anal..

[20] Serce S., Ercisli S., Sengul M., Gunduz K., Orhan E. (2010). Antioxidant Activities and Fatty Acid Composition of Wild Grown Myrtle (*Myrtus communis* L.) Fruits. Pharmacogn. Mag..

[21] Azman E.M., Yusof N., Chatzifragkou A., Charalampopoulos D. (2022). Stability Enhancement of Anthocyanins from Blackcurrant (*Ribes nigrum* L.) Pomace through Intermolecular Copigmentation. Molecules.

[22] Garzón G.A., Soto C.Y., López-R M., Riedl K.M., Browmiller C.R., Howard L. (2020). Phenolic Profile, in Vitro Antimicrobial Activity and Antioxidant Capacity of Vaccinium Meridionale Swartz Pomace. Heliyon.

[23] Aliaño González M.J., Carrera C., Barbero G.F., Palma M. (2022). A Comparison Study between Ultrasound–Assisted and Enzyme–Assisted Extraction of Anthocyanins from Blackcurrant (*Ribes nigrum* L.). Food Chem. X.

[24] Krithika J.S., Sathiyasree B., Beniz Theodore E., Chithiraikannu R., Gurushankar K. (2022). Optimization of Extraction Parameters and Stabilization of Anthocyanin from Onion Peel. Crit. Rev. Food Sci. Nutr..

[25] Kang H.-J., Ko M.-J., Chung M.-S. (2021). Anthocyanin Structure and PH Dependent Extraction Characteristics from Blueberries (*Vaccinium corymbosum*) and Chokeberries (*Aronia melanocarpa*) in Subcritical Water State. Foods.

[26] Bonfigli M., Godoy E., Reinheimer M.A., Scenna N.J. (2017). Comparison between Conventional and Ultrasound-Assisted Techniques for Extraction of Anthocyanins from Grape Pomace. Experimental Results and Mathematical Modeling. J. Food Eng..

[27] Rodríguez L., Plaza A., Méndez D., Carrasco B., Tellería F., Palomo I., Fuentes E. (2022). Antioxidant Capacity and Antiplatelet Activity of Aqueous Extracts of Common Bean (*Phaseolus Vulgaris* L.) Obtained with Microwave and Ultrasound Assisted Extraction. Plants.

[28] Albuquerque B.R., Oliveira M.B.P.P., Barros L., Ferreira I.C.F.R. (2020). Could Fruits Be a Reliable Source of Food Colorants? Pros and Cons of These Natural Additives. Crit. Rev. Food Sci. Nutr..

[29] Chemat F., Rombaut N., Sicaire A.-G., Meullemiestre A., Fabiano-Tixier A.-S., Abert-Vian M. (2017). Ultrasound Assisted Extraction of Food and Natural Products. Mechanisms, Techniques, Combinations, Protocols and Applications. A Review. Ultrason. Sonochem..

[30] Pereira P., Cebola M.-J., Oliveira M.C., Bernardo Gil M.G. (2017). Antioxidant Capacity and Identification of Bioactive Compounds of *Myrtus communis* L. Extract Obtained by Ultrasound-Assisted Extraction. J. Food Sci. Technol..

[31] Bouaoudia-Madi N., Boulekbache-Makhlouf L., Kadri N., Dahmoune F., Remini H., Dairi S., Oukhmanou-Bensidhoum S., Madani K. (2017). Phytochemical Analysis of *Myrtus communis* Plant: Conventional versus Microwave Assisted-Extraction Procedures. J. Complement. Integr. Med..

[32] González de Peredo V.A., Vázquez-Espinosa M., Espada-Bellido E., Jiménez-Cantizano A., Ferreiro-González M., Amores-Arrocha A., Palma M., G. Barroso C., F. Barbero G. (2018). Development of New Analytical Microwave-Assisted Extraction Methods for Bioactive Compounds from Myrtle (*Myrtus communis* L.). Molecules.

[33] AOAC (2005). Official Methods of Analysis.

[34] Loughrey K. (2002). Overview of Color Analysis. Current Protocols in Food Analytical Chemistry.

[35] Giusti M.M., Wrolstad R.E. (2001). Characterization and Measurement of Anthocyanins by UV-Visible Spectroscopy. Curr. Protoc. Food Anal. Chem..

[36] Vega E.N., García-Herrera P., Ciudad-Mulero M., Dias M.I., Matallana-González M.C., Cámara M., Tardío J., Molina M., Pinela J., Pires T.C.S.P. (2023). Wild Sweet Cherry, Strawberry and Bilberry as Underestimated Sources of Natural Colorants and Bioactive Compounds with Functional Properties. Food Chem..

[37] Gonçalves G.A., Soares A.A., Correa R.C.G., Barros L., Haminiuk C.W.I., Peralta R.M., Ferreira I.C.F.R., Bracht A. (2017). Merlot Grape Pomace Hydroalcoholic Extract Improves the Oxidative and Inflammatory States of Rats with Adjuvant-Induced Arthritis. J. Funct. Foods.

[38] Vega E.N., Fernández-Ruiz V., Sánchez-Mata M.C., Cámara M., Morales P. (2024). A Rapid and Simple UHPLC- DAD Method for Individual Anthocyanin Analysis: Optimization and Validation in Wild Mediterranean Berries. Food Anal. Methods.

[39] Medina M.B. (2011). Determination of the Total Phenolics in Juices and Superfruits by a Novel Chemical Method. J. Funct. Foods.

[40] Palombini S.V., Claus T., Maruyama S.A., Carbonera F., Montanher P.F., Visentainer J.V., Gomes S.T.M., Matsushita M. (2016). Optimization of a New Methodology for Determination of Total Phenolic Content in Rice Employing Fast Blue BB and QUENCHER Procedure. J. Braz. Chem. Soc..

[41] Bonoli M., Verardo V., Marconi E., Caboni M.F. (2004). Antioxidant Phenols in Barley (*Hordeum Vulgare* L.) Flour: Comparative Spectrophotometric Study among Extraction Methods of Free and Bound Phenolic Compounds. J. Agric. Food Chem..

[42] Hacıseferoğulları H., Özcan M.M., Arslan D., Ünver A. (2012). Biochemical Compositional and Technological Characterizations of Black and White Myrtle (*Myrtus communis* L.) Fruits. J. Food Sci. Technol..

[43] Çelik B., Şan B. (2023). Determination of Biochemical Contents of Myrtle (*Myrtus communis* L.) Fruits at Different Maturity Levels. Erwerbs-Obstbau.

[44] Aydın C., Özcan M.M. (2007). Determination of Nutritional and Physical Properties of Myrtle (*Myrtus communis* L.) Fruits Growing Wild in Turkey. J. Food Eng..

[45] Mulas M., Fadda A., Angioni A. (2013). Effect of Maturation and Cold Storage on the Organic Acid Composition of Myrtle Fruits. J. Sci. Food Agric..

[46] González de Peredo A.V., Vázquez-Espinosa M., Espada-Bellido E., Ferreiro-González M., Amores-Arrocha A., Palma M., Barbero G.F., Jiménez-Cantizano A. (2019). Alternative Ultrasound-Assisted Method for the Extraction of the Bioactive Compounds Present in Myrtle (*Myrtus communis* L.). Molecules.

[47] Sik B., Ajtony Z., Lakatos E., Székelyhidi R. (2024). Wild Blackberry Fruit (*Rubus Fruticosus* L.) as Potential Functional Ingredient in Food: Ultrasound-Assisted Extraction Optimization, Ripening Period Evaluation, Application in Muffin, and Consumer Acceptance. Foods.

[48] Wu X., Prior R.L. (2005). Systematic Identification and Characterization of Anthocyanins by HPLC-ESI-MS/MS in Common Foods in the United States: Fruits and Berries. J. Agric. Food Chem..

[49] Messaoud C., Boussaid M. (2011). *Myrtus communis* Berry Color Morphs: A Comparative Analysis of Essential Oils, Fatty Acids, Phenolic Compounds, and Antioxidant Activities. Chem. Biodivers..

[50] López-Froilán R., Hernández-Ledesma B., Cámara M., Pérez-Rodríguez M.L. (2018). Evaluation of the Antioxidant Potential of Mixed Fruit-Based Beverages: A New Insight on the Folin-Ciocalteu Method. Food Anal. Methods.

[51] Monari S., Ferri M., Salinitro M., Tassoni A. (2023). New Insights on Primary and Secondary Metabolite Contents of Seven Italian Wild Food Plants with Medicinal Applications: A Comparative Study. Plants.

[52] Barboni T., Cannac M., Massi L., Perez-Ramirez Y., Chiaramonti N. (2010). Variability of Polyphenol Compounds in *Myrtus communis* L. (Myrtaceae) Berries from Corsica. Molecules.

[53] Castrejón A.D.R., Eichholz I., Rohn S., Kroh L.W., Huyskens-Keil S. (2008). Phenolic Profile and Antioxidant Activity of Highbush Blueberry (*Vaccinium Corymbosum* L.) during Fruit Maturation and Ripening. Food Chem..

[54] Sójka M., Kołodziejczyk K., Milala J. (2013). Polyphenolic and Basic Chemical Composition of Black Chokeberry Industrial By-Products. Ind. Crops Prod..

[55] Kähkönen M.P., Hopia A.I., Heinonen M. (2001). Berry Phenolics and Their Antioxidant Activity. J. Agric. Food Chem..

